# Regulation of neuroblast proliferation by surface glia in the *Drosophila* larval brain

**DOI:** 10.1038/s41598-018-22028-y

**Published:** 2018-02-27

**Authors:** Makoto I. Kanai, Myung-Jun Kim, Takuya Akiyama, Masahiko Takemura, Kristi Wharton, Michael B. O’Connor, Hiroshi Nakato

**Affiliations:** 1 Department of Genetics, Cell Biology and Development, University of Minnesota, Minneapolis, MN 55455 USA; 2 Department of Molecular Biology, Cell Biology and Biochemistry, Brown University, Providence, RI 02912 USA

## Abstract

Despite the importance of precisely regulating stem cell division, the molecular basis for this control is still elusive. Here, we show that surface glia in the developing *Drosophila* brain play essential roles in regulating the proliferation of neural stem cells, neuroblasts (NBs). We found that two classes of extracellular factors, Dally-like (Dlp), a heparan sulfate proteoglycan, and Glass bottom boat (Gbb), a BMP homologue, are required for proper NB proliferation. Interestingly, Dlp expressed in perineural glia (PG), the most outer layer of the surface glia, is responsible for NB proliferation. Consistent with this finding, functional ablation of PG using a dominant-negative form of dynamin showed that PG has an instructive role in regulating NB proliferation. Gbb acts not only as an autocrine proliferation factor in NBs but also as a paracrine survival signal in the PG. We propose that bidirectional communication between NBs and glia through TGF-β signaling influences mutual development of these two cell types. We also discuss the possibility that PG and NBs communicate via direct membrane contact or transcytotic transport of membrane components. Thus, our study shows that the surface glia acts not only as a simple structural insulator but also a dynamic regulator of brain development.

## Introduction

The *Drosophila* CNS develops from neural stem cells called neuroblasts (NBs)^1–3^. The self-renewal and differentiation of NBs are thought to be controlled by both intrinsic programs in the NBs as well as extrinsic cues. Despite a wealth of knowledge about intrinsic factors that regulate NB development^4^, very little is known regarding non-autonomous factors that affect this process. In the embryonic CNS, it has been shown that NBs receive an extrinsic signal from the overlying epithelium to properly orient the mitotic spindle axis^5^. Several lines of evidence suggest that post-embryonic NBs are also controlled by extrinsic factors. One previous study showed that the activin receptor, Baboon (Babo), and its transcriptional mediator, Smad2 (Smox), regulate NB proliferation in the larval CNS, indicating that activin-like ligands may play a role in this process^6^. Furthermore, expression of a dominant-negative form of E-cadherin in glia reduced the number of proliferating NBs^7^. This observation indicated that physical contact of larval NBs with glia is critical to NB proliferation. Larval glia are also known to secrete Anachronism, which maintains NBs in a mitotically inactive state (the quiescent state) during early larval stages^8^. Thus, although there is no direct evidence that *Drosophila* larval glia have a niche function, it is assumed that division and differentiation of larval brain NBs may be controlled by surrounding glial cells.

Recent studies demonstrated vital roles of heparan sulfate proteoglycans (HSPGs) as important regulators of stem cell behaviors^9–14^. HSPGs are a class of carbohydrate-modified proteins involved in a variety of biological processes such as growth factor signaling, cell adhesion, and enzymatic catalysis^15^. Importantly, these molecules serve as co-receptors for growth factor signaling, regulating reception and tissue distribution of secreted signaling proteins such as FGFs, BMPs, Wnts, and Hedgehog on the cell surface^16–21^. Although HSPGs are generally thought to regulate growth factor signaling on the surface of signal-receiving cells, they can also activate signaling *in trans* from adjacent cells to play non-autonomous roles^9,11,22,23^.

The TGF-β pathway is an evolutionarily conserved signal transduction module that mediates diverse biological processes in animals, including stem cell control. Like in mammals, both the BMP and Activin branches are required for *Drosophila* development and homeostasis^24^. BMPs are involved in numerous processes throughout all developmental stages, from patterning of the embryo^25,26^ and imaginal discs^27,28^ to stem cell maintenance^29^. Activin branch ligands also play diverse roles in growth and patterning in *Drosophila*, including control of imaginal cell proliferation^30–32^, neuroblast proliferation^6^, neuron remodeling^33,34^, axon guidance^35,36^, and sugar homeostasis^37^.

Glial cells in the *Drosophila* larval brain have been classified into a few different types based on their positions, morphology, and gene expression profiles, including perineural glia (PG), subperineural glia (SPG), cortex glia, and astrocyte-like glia, which send processes into the neuropil^38–40^. Two surface glia cell types, PG and SPG, provide a structural basis for an insulation barrier between the CNS and hemolymph known as the blood-brain barrier (BBB)^41–43^. The most outer layer is formed by PG, which is characterized by the formation of extensive, thin cell protrusions. The SPG are located immediately below the PG. The SPG forms septate junctions (SJs) and are central for the *Drosophila* BBB function^44,45^. The BBB restricts molecular movement at the interface between blood and nervous tissues, and it plays critical roles in maintaining a regulated microenvironment for reliable neural signaling^46,47^. On the other hand, the BBB is a major obstacle for pharmacologic treatments to the brain. Past studies have focused on the functions of surface glia as an effective shield, which separates the nervous system from the open circulatory system, to maintain brain homeostasis^42^. However, the biological role of surface glia in regulating neural development remains to be elucidated.

In this study, we identified bidirectional interactions of NBs and surface glial cells in the developing larval brain. We found that Dally-like (Dlp), a HSPG of the glypican family, is expressed in PG and is critical for proper NB proliferation. Selective ablation of glial cells supported the idea that PG play an instructive role in regulating NB proliferation. We also found that Glass bottom boat (Gbb), a BMP homologue, is expressed in NBs and functions with Dlp to regulate normal proliferation of NBs. In addition to serving as an autocrine signal in NBs, Gbb also acts as a paracrine survival signal in the PG. Thus, the development of NBs and glia is controlled by reciprocal communication between these cells through TGF-β signaling pathways in the NSC niche. Finally, we discuss the possibility that PG make physical membrane contact with NBs.

## Results

### Dlp is required for normal NB proliferation

**Figure 1:**
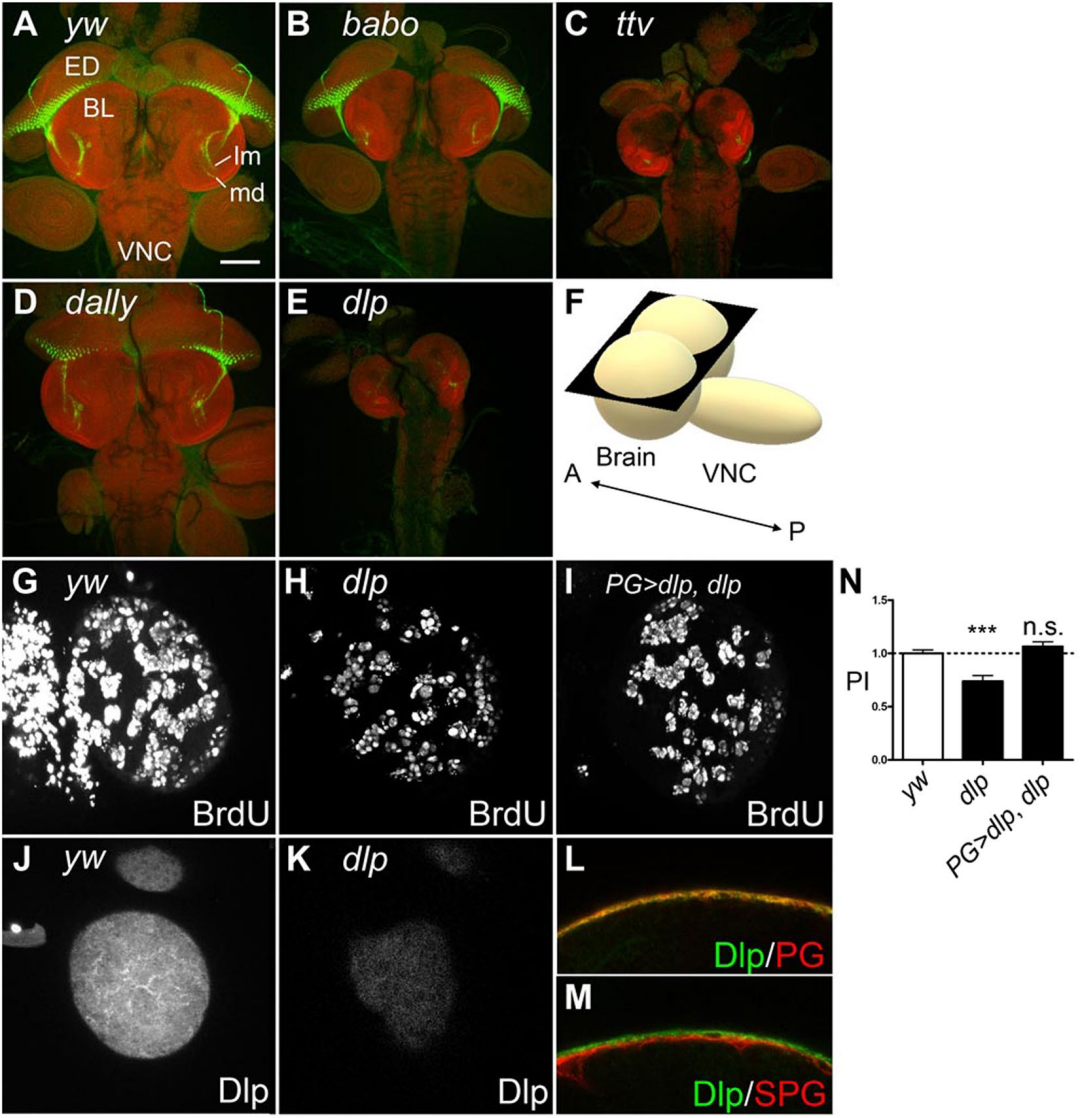
*dlp* regulates NB proliferation in *Drosophil*a larval brain. (**A–E**) Morphology of brains from control (*yw*) (**A**), and *babo*^*Fd4*^ (**B**), *ttv*^*524*^ (**C**), *dally*^*gem*^ (**D**) and *dlp*^*1*^ (**E**) mutants. Photoreceptor (PR) axon projections are assessed by 24B10 staining (green). Propidium iodide (red) staining shows brain lobe (BL), eye disc (ED) and ventral nerve cord (VNC). In control and *dally* brains, the PR axons form a neural plexus at the lamina (lm) and project to the medulla (md). *babo*, *ttv* and *dlp* brains show reduced brain size and abnormally bundled growth cones. Although most of *dlp*^*1*^ homozygous mutants die during embryogenesis, we obtained rare survivors (**E**). Anterior to the top. Scale bar: 100 µm. (**F**) A schematic illustration of *Drosophila* larval brain and ventral nerve cord (VNC). A and P shows anterior-posterior axis. Most images in this paper are horizontal confocal optic sections (black plate) unless otherwise stated. (**G**–**I**) BrdU incorporation into NBs in control (**G**) and *dlp*^*1*^*/dlp*^*A*1*87*^ (**H**), and *PG-Gal4 dlp*^*A*1*87*^*/UAS-dlp dlp*^*1*^ (**I**). NB proliferation was impaired in *dlp* mutants. *dlp* expression in PG fully rescued the NB proliferation defect of *dlp* mutants. (**J** and **K**) Expression of Dlp. Anti-Dlp antibody staining of *yw* (**J**) and *dlp*^*1*^*/dlp*^*A187*^ (**K**) brains. Images are shown for surface glial cell focal plane. Dlp is detected on the surface of the brain lobe largely overlapping with PG. Loss of the signal confirmed the specificity of anti-Dlp staining. (**L** and **M**) Cross-sections of brains from *PG* > *mCD8ChRFP* (**L**) and *SPG* > *mCD8ChRFP* (**M**) larvae stained with anti-Dlp antibody. Signals for anti-Dlp and mCD8ChRFP are shown red and green, respectively. (**N**) Summary of PI for each genotype. Numerical figures depict the mean ± SE. In this, and the following figures, data were analyzed by one-way ANOVA with Bonferroni post hoc test. n.s.: not significant; ***p < 0.001.

HS-deficient mutants, *tout velu* (*ttv*) and *sulfateless*, show a small brain phenotype mainly because the sizes of brain lobes and ventral ganglion do not properly increase during third larval instar stage. A previous study showed that mutations in activin signaling components cause a similar small brain phenotype, which is associated with altered innervation of photoreceptor (PR) axons (Fig. 1B)^6^. The PR axon innervation abnormality is due to a reduction in the size of PR target field in the CNS, the lamina and medulla, rather than defects of PR cells. Thus, PR axon projection is a sensitive readout of brain growth. Similar to a mutant for *baboon* (*babo*), the *Drosophila* activin type I receptor, *ttv* mutant brains show PR axon targeting defect revealed by staining with anti-Chaoptin antibody (22B10) (Fig. 1C). This observation suggests that one or more HSPG molecules are required for normal NB proliferation. No obvious small brain phenotype is caused by mutations in *dally*, one of two integral-membrane HSPGs of the glypican type (Fig. 1D), or *dSyndecan*, the only *Drosophila* Syndecan homologue (data not shown). We found that brains mutant for *dlp*, the second glypican homologue, exhibit a very severe small brain phenotype with abnormalities of PR axon projection (Fig. 1E), suggesting that *dlp* is required for normal brain growth.

To determine if the small brain phenotype of *dlp* mutants is caused by reduced proliferation of NBs, we analyzed NB proliferation in control and mutant brains by BrdU labeling (Fig. 1G–I). In control (*yw*) third instar larval brains, where NBs are actively dividing, BrdU incorporation showed a unique and robust pattern in NBs and their progeny (Fig. 1G). Although *dlp* null mutants have been described as embryonic lethal, trans allelic heterozygotes of certain *dlp* loss-of-function mutations (*dlp*^*1*^*/dlp*^*A*187^) survive to late third larval instar stage. We found that *dlp*^*1*^*/dlp*^*A187*^ mutant brain shows significantly impaired NB proliferation (Fig. 1H and N; Proliferation Index (PI) = 0.74).

Interestingly, Dlp is predominantly, although not exclusively, expressed at the surface of larval brains (Fig. 1J and K). To further define the localization of Dlp on the brain surface, we used well-established surface glia Gal4 drivers, *NP6293-Gal*4 and *NP2276-Gal4*. We carefully assessed the expression patterns of these Gal4 drivers and confirmed that *NP6293-Gal4* and *NP2276-Gal4* are specifically expressed in PG and SPG, respectively (Fig. S1, data not shown), consistent with previous studies. Therefore, hereafter, we will call these drivers *PG-Gal4* and *SPG-Gal4*, respectively, in this paper. To define Dlp-expressing cells, fluorescent UAS transgene reporters were induced to mark glia membranes using *PG-Gal4* and *SPG-Gal4*. Anti-Dlp antibody staining of brains from *PG* > *mCD8ChRFP* (animals bearing *PG-Gal4* and *UAS-mCD8ChRFP*) and *SPG* > *mCD8ChRFP* showed that the area of Dlp expression is largely overlapping with PG (Fig. 1L and M).

To determine if Dlp on the surface glia non-autonomously regulates NB development, we specifically expressed *dlp* in PG of *dlp* mutants using *PG-Gal4*. We found that the reduced NB proliferation of *dlp* mutants was completely restored by PG-specific expression of *dlp* (Fig. 1I and N; PI = 1.07). Since no leaky expression of *PG-Gal4* is detectable in NBs or any parts of the brain inside the surface glia (Fig. S1), this rescue experiment demonstrated that Dlp expressed in PG can sufficiently function to maintain normal NB proliferation.

### Perineural glia are required for normal development of NBs

**Figure 2:**
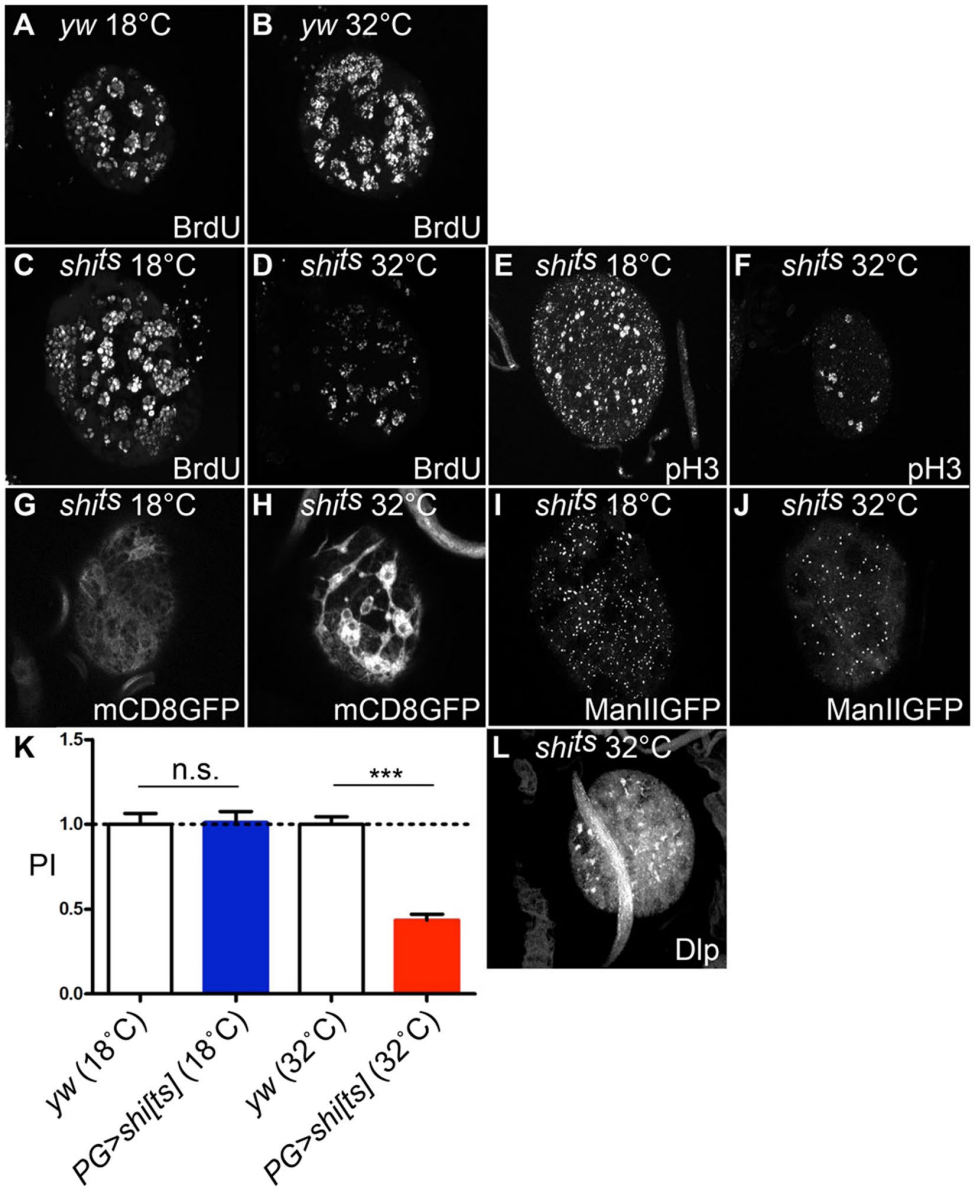
Functional ablation of PG caused a NB proliferation defect. (**A**–**F**) Blocking *shibire* disrupts normal proliferation of NBs. BrdU incorporation in control (**A** and **B**) and *PG* > *shi*^*ts*^ (**C** and **D**) brains at permissive (18 °C; **A** and **C**) and restrictive (32 °C; **B** and **D**) temperatures. (**E** and **F**) *shi*^*ts*^ expression reduces pH3-positive M phase cells. Anti-pH3 staining of *PG* > *shi*^*ts*^ at permissive (**E**) and restrictive (**F**) temperatures. (**G**–**J**) *shi*^*ts*^ expression affects membrane distribution. Membrane distribution was visualized in *PG-Gal*4/+*; UAS-mCD8GFP/UAS-shi*^*ts*^ at permissive (**G**) and restrictive (**H**) temperatures. The localization of ManII-eGFP was examined in *PG-Gal4*/+*; UAS-ManII-eGFP/UAS-shi*^*ts*^ at permissive (**I**) and restrictive (**J**) temperatures. (**K**) Summary of PI for each genotype. (**L**) Dlp localization in *PG* > *shi*^*ts*^ brains at restrictive temperature. *shi*^*ts*^ expression disrupts cell surface localization of Dlp. This image includes tissue debris with unknown origin on the top of the brain. n.s.: not significant; ***p < 0.001.

To examine the role of PG in NB development, we tested the effects of functionally ablating glial cells *in vivo* using a temperature-sensitive dominant-negative form of *shibire* (*shi*), the *Drosophila* dynamin^48^. Since dynamin is required for the formation of membrane vesicles^49^, expression of this dominant negative form of *shi* (*shi*^*ts*^) can block various types of membrane trafficking events, including endocytosis and protein secretion, and has been effectively used to disrupt glial cell function^50–52^. We expressed *UAS-shi*^*ts*^ specifically in PG using *PG-Gal4* (*PG* > *shi*^*ts*^) and assessed its effect on NB proliferation. We found that the functional ablation of PG by expression of *shi*^*ts*^ severely disrupted NB cell proliferation (Fig. 2D and K; PI = 1.01 and 0.43 at permissive (18 °C) and restrictive (32 °C) temperatures, respectively), which resulted in a small brain phenotype (data not shown). Normal NB proliferation was observed in control (*yw*) brains for both temperature conditions (Fig. 2A and B). *PG* > *shi*^*ts*^ reared at 32 °C also showed a decreased number of M-phase cells labeled with anti-phosphorylated histone H3 (pH3) (Fig. 2E and F). Brains from various control animals (*PG* > *shi*^*ts*^ reared at the permissive temperature, *UAS* > *shi*^*ts*^ without Gal4 driver) were indistinguishable from wild-type (Fig. 2C and E, data not shown). These observations show that functional ablation of PG disrupts normal proliferation of neighboring NBs.

To further explore the cellular and molecular basis for the role of PG in NB proliferation, we examined the effect of *shi*^*ts*^ on membrane structures in PG. We observed a gross abnormality in patterns of a membrane-bound form of GFP (mCD8GFP) in *PG* > *shi*^*ts*^ at the restrictive temperature (Fig. 2G and H). This cell membrane marker accumulated in restricted areas, forming patches, and failed to uniformly distribute on the PG plasma membrane. Expression of *shi*^*ts*^ in PG also affected a marker for the Golgi (ManII-eGFP) (Fig. 2I and J). Altered patterns of these membrane markers suggested that normal membrane trafficking is required for proper control of NB development.

We next asked if Dlp localization is affected by *shi*^*ts*^ expression. In a section near the brain surface, the PG focal plane, anti-Dlp staining showed abnormal aggregations of Dlp-containing membrane in *PG* > *shi*^*ts*^ brain at 32 °C (Fig. 2L, compare to Fig. 1J). This observation suggests that the mislocalization of Dlp in the PG cell membrane may contribute to the NB proliferation defect of *shi*^*ts*^ brains. Altogether, these results support the idea that Dlp on the PG surface regulates NB proliferation.

### Gbb signaling regulates NB proliferation

**Figure 3:**
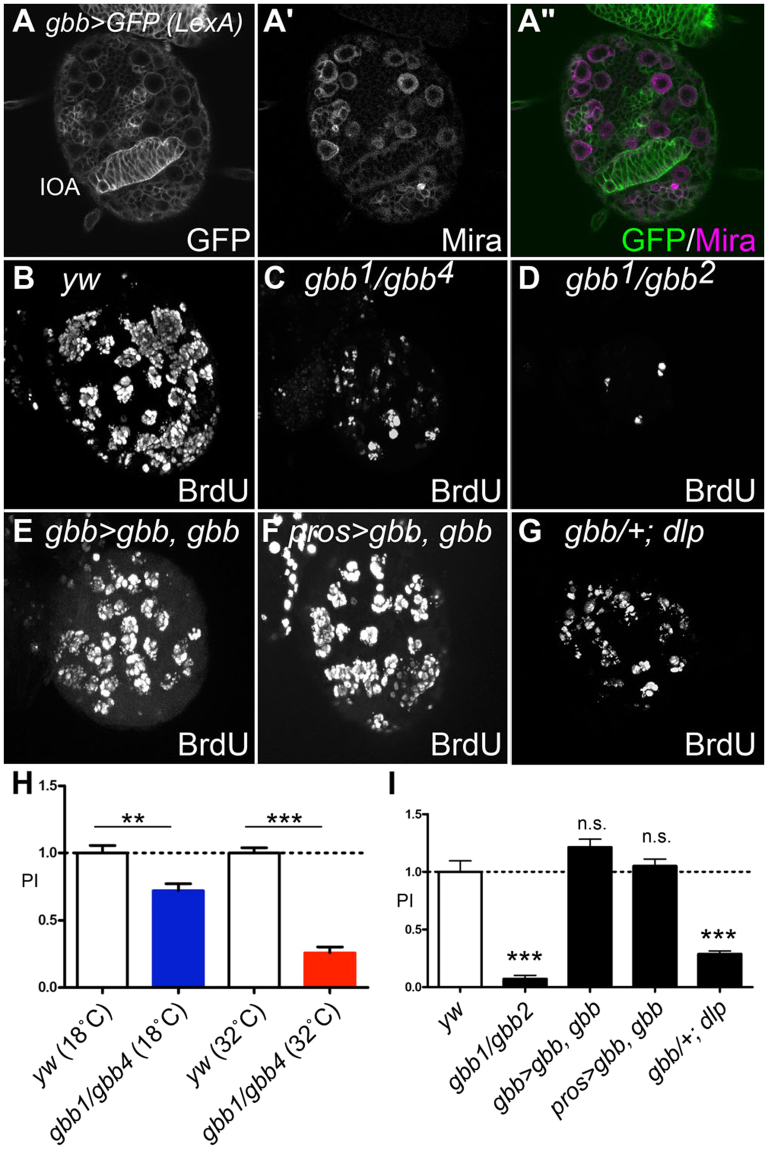
*gbb* controls NB proliferation. (**A**–**A”**) *gbb* is expressed in NBs. Expression of *gbb-LexAVP*1*6* was monitored by *LexAop-rCD*2*GFP* (A) and NBs are labeled with anti-Mira antibody (**A’**). (**A”**) Merged image of **A** and **A’**. IOA: inner optic anlage. (**B**–**G**) BrdU incorporation of control (*yw* reared at 32 °C; B), *gbb*^*1*^*/gbb*^*4*^ (reared at 32 °C; **C**), *gbb*^*1*^*/gbb*^*2*^ (**D**), *gbb*^*1*^ *gbb-Gal4/gbb*^*2*^ *UAS-gbb* (**E**), *gbb*^*1*^ *pros-Gal4/gbb*^*2*^ *UAS-gbb* (**F**), and *gbb*^*1*^/+*; dlp*^*1*^*/dlp*^*A187*^ (**G**) brains. NB proliferation was severely disrupted in *gbb* mutants. This phenotype is rescued by *gbb* expression in NBs. Heterozygosity for *gbb* enhanced the NB proliferation defect of *dlp* mutants, suggesting that these two genes function in the same pathway. (**H** and **I**) Summary of PI for each genotype. Numerical figures depict the mean ± SE. n.s.: not significant; **p < 0.01; ***p < 0.001.

The requirement for Dlp in NB proliferation suggests that a signaling molecule controls NB development in a HSPG-dependent manner. Previous studies have shown that most HS-dependent ligands are dispensable for NB development at the third instar larval stage^53,54^, or they are not expressed in the corresponding regions^54–56^. An exception is Gbb, a BMP-like ligand, which has been found to be expressed in the developing larval CNS by RNA *in situ* hybridization^57^. We monitored *gbb* expression at higher resolution, using *gbb-lacZ* and *gbb-LexA* > *LexAop-rCD2GFP*, in which *gbb* enhancers drive expression of a membrane-bound form of GFP, to determine the specific cell types that express *gbb*. As shown in Fig. 3A, a reporter assay revealed that *gbb* expression overlaps with a NB-marker, Mira. We also asked if *gbb* is expressed in PG. In this and subsequent experiments, we used an antibody against a nuclear receptor Seven-up (Svp) as a specific marker for PG since we have found Svp expression extensively overlaps with *PG-Gal4*-positive cells (Fig. S1B-B”). Thus, anti-Svp is a useful tool to label PG. We observed no overlap in expression between *gbb-lacZ* and Svp (Fig. S2). Thus, *gbb* is specifically expressed in the NBs but not in PG.

We next examined whether *gbb* is required for NB proliferation. Blocking *gbb* activity at the third instar using a temperature-sensitive allelic combination (*gbb*^*1*^*/gbb*^*4*^) severely disrupted NB proliferation (Fig. 3C and H; PI = 0.72 at permissive temperature, 0.26 at restrictive temperature). An even more severe NB division defect was observed in the early third instar survivors of *gbb* null (*gbb*^*1*^*/gbb*^*2*^) mutants (Fig. 3D and I; PI = 0.07). This *gbb*^*1*^*/gbb*^*2*^ phenotype was fully rescued by induction of *UAS-gbb* using either *gbb-Gal4* or *pros-Gal4*, another NB-specific driver (Fig. 3E,F and I; PI = 1.21 and 1.05, respectively). These results confirmed that Gbb produced in NBs is responsible for normal cell proliferation of NBs (Fig. 3E and F).

To determine if *gbb* and *dlp* function in the same pathway to regulate NB development, we examined genetic interactions between the two genes. The NB proliferation defect of *dlp* mutants was significantly aggravated by heterozygosity for *gbb* in *gbb*^*1*^/+*; dlp*^*1*^*/dlp*^*A*1*87*^ brains (Fig. 3G and I, compare to Fig. 1B; PI = 0.29), while *gbb* heterozygotes (*gbb*^*1*^/+) show normal NB proliferation (data not shown). This synergistic effect is consistent with the notion that the *gbb* and *dlp* genes function in the same pathway to regulate NB proliferation.

### Role of Gbb autocrine signaling in NB proliferation

**Figure 4:**
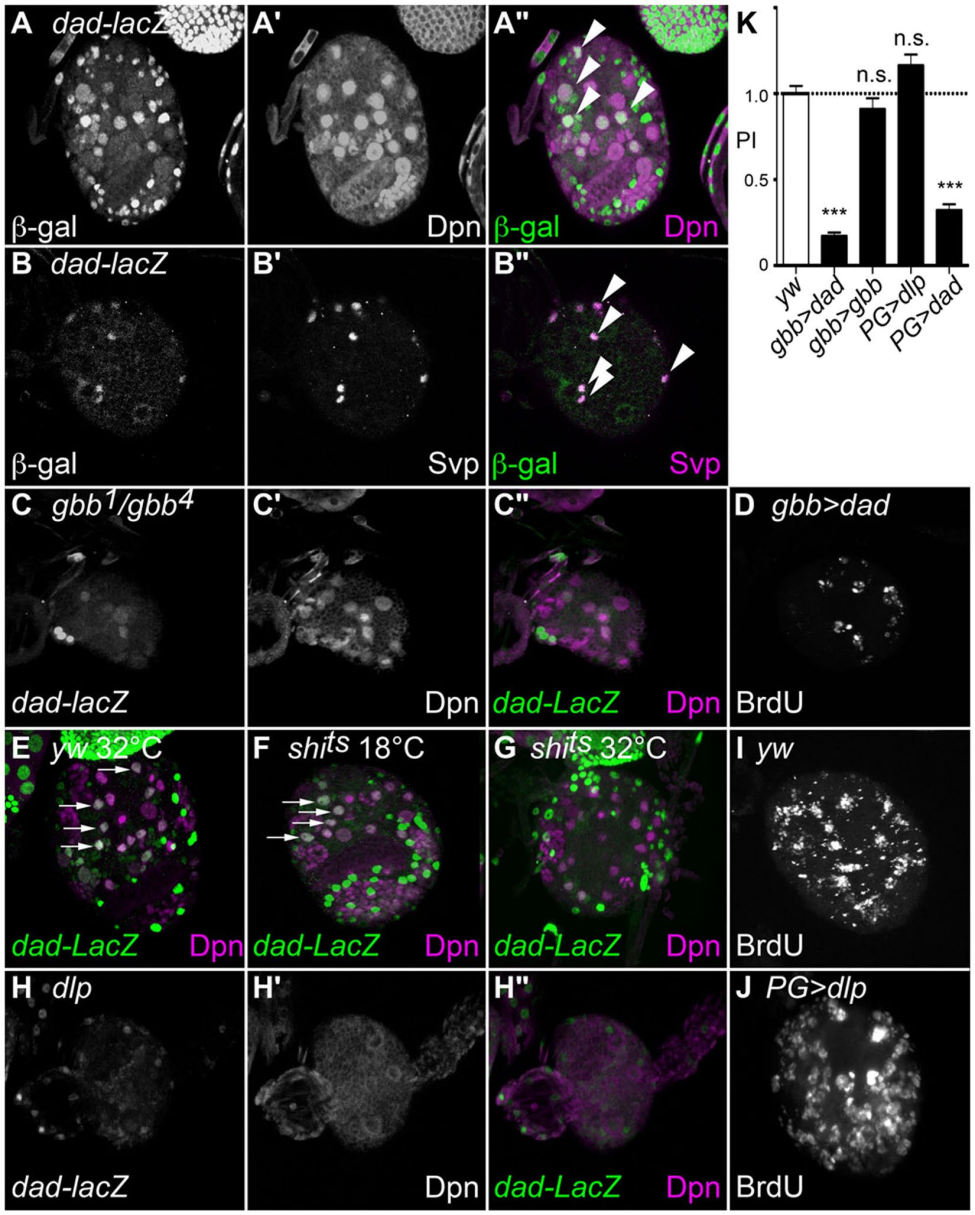
Gbb autocrine signaling in NB proliferation. (**A–B”**) *dad-lacZ*/+ brains were stained with anti-β-galactosidase (**A** and **B**) and anti-Dpn (a NB marker; **A’**) or anti-Svp (a PG marker; **B’**) antibodies. (**A”** and **B”**) are merged images of A and A’, B and B’, respectively. Arrows show overlapped expression of *dad-lacZ* with Dpn (**A”**) and Svp (**B”**). *dad-lacZ* expression was detected in both NBs and PG. (**C–C”**) *dad-lacZ* expression (**C**) and anti-Dpn (**C’**) staining in *gbb*^*1*^*/gbb*^*4*^ (reared at 32 °C) brain. C” is merged image of C and C’. *dad-lacZ* signal was significantly diminished in *gbb* mutants. (**D**) BrdU incorporation of *gbb-Gal4/UAS-dad* brain. (**E**-**G**) *dad-lacZ* expression (green) and anti-Dpn staining (magenta) in control (**E**), *PG> shibire*^*ts*^ (**F** and **G**) brains at permissive (**F**) and restrictive (**E** and **G**) temperatures. Gbb signaling is impaired by PG functional ablation. (**H–H”**) *dad-lacZ* expression (**H**) and anti-Dpn staining (H’) of *dlp*^*1*^*/dlp*^*A*1*87*^ brain. H” is a merged image of H and H’. The number of *dad-lacZ*-positive NBs was significantly decreased in *dlp* mutants. (**I** and **J**) BrdU incorporation in control (*yw*; I) and *PG-Gal4/UAS-dlp* (**J**) brains reared at 29 °C. (**K**) Summary of PI for each genotype. n.s.: not significant; ***p < 0.001.

We next asked which cells in the developing brain respond to the Gbb signal using *dad-lacZ*^58^, a widely-used reporter of BMP signaling activity. Interestingly, we detected specific *dad-lacZ* signals in both Dpn-positive NBs and Svp-positive PG (Fig. 4A–B”), showing that Gbb signaling is received by both NBs and PG. Most *dad-lacZ* expression was lost in *gbb* mutant brains (Fig. 4C–C”). This result indicated that *gbb* is responsible for most, if not all, BMP signaling in this region of the brain at third instar larval stage. When we specifically blocked Gbb signaling in NBs by using *gbb-Gal*4 to express *UAS-dad*, an inhibitor of BMP signaling, NB proliferation was strongly inhibited (Fig. 4D and K; PI = 0.17). Since surrounding glia are wild-type, this result demonstrated an autonomous role for Gbb signaling in NB proliferation.

If PG support NB proliferation through activation and/or maintenance of Gbb signaling, one could expect that *shi*^*ts*^ expression in PG would impair Gbb signaling in NBs. We found that this was the case. Gbb signaling in NBs can be monitored by co-expression of *dad-lacZ* and Dpn (arrows in Fig. 4E and F). Expression of *dad-lacZ* in Dpn-positive cells was eliminated by PG-specific expression of *shi*^*ts*^ (Fig. 4G). In addition, we also observed that *dad-lacZ* signal was diminished in *dlp* mutants (Fig. 4H–H”). Together with the genetic interactions between *gbb* and *dlp* shown above, these results indicated that Dlp in the PG potentiates autocrine Gbb signaling in NB to promote its proliferation. We also evaluated the effects of *gbb* or *dlp* overexpression on the NB phenotype, but neither caused any significant change in NB proliferation (Fig. 4K; PI = 0.91 and 1.16, respectively).

We have previously shown that Dlp acts as a co-receptor for Gbb *in vitro* using a *trans* signaling assay^11^. This assay measures the activity of a co-receptor expressed in a contacting neighboring cell to regulate signaling *in trans* in the signal-receiving cell. This *in vitro* data supports our model that Dlp (on PG surface) can act as a *trans* co-receptor for Gbb (received by NB) in the brain. Since no previous work has shown a direct binding between Dlp and Gbb, we attempted to show Dlp-Gbb complex formation by co-immunoprecipitation of the proteins expressed in S2 cells. However, the Gbb-Dlp complex was not detectable, probably because the level of HS modification onto the Dlp core-protein in S2 cells is too low. As shown in Fig. S3A and a previous work^59^, Gbb binds to heparin-sepharose beads. Thus, similar to many other BMP members, Gbb is a heparin-binding protein^59^, characteristic of HS-dependent factors.

We also analyzed the cellular localization of Gbb and Dlp by expressing Gbb-HA and Dlp-Myc in S2 cells. Immunotaining with anti-HA and anti-Myc antibodies using an extracellular staining protocol revealed that Gbb and Dlp extensively co-localize on the S2 cell surface (Fig. S3B). Together, our results and previous reports suggest that Dlp serves as a co-receptor for Gbb signaling to regulate NB proliferation in the larval brain.

### Role of Gbb signaling in perineural glia

**Figure 5:**
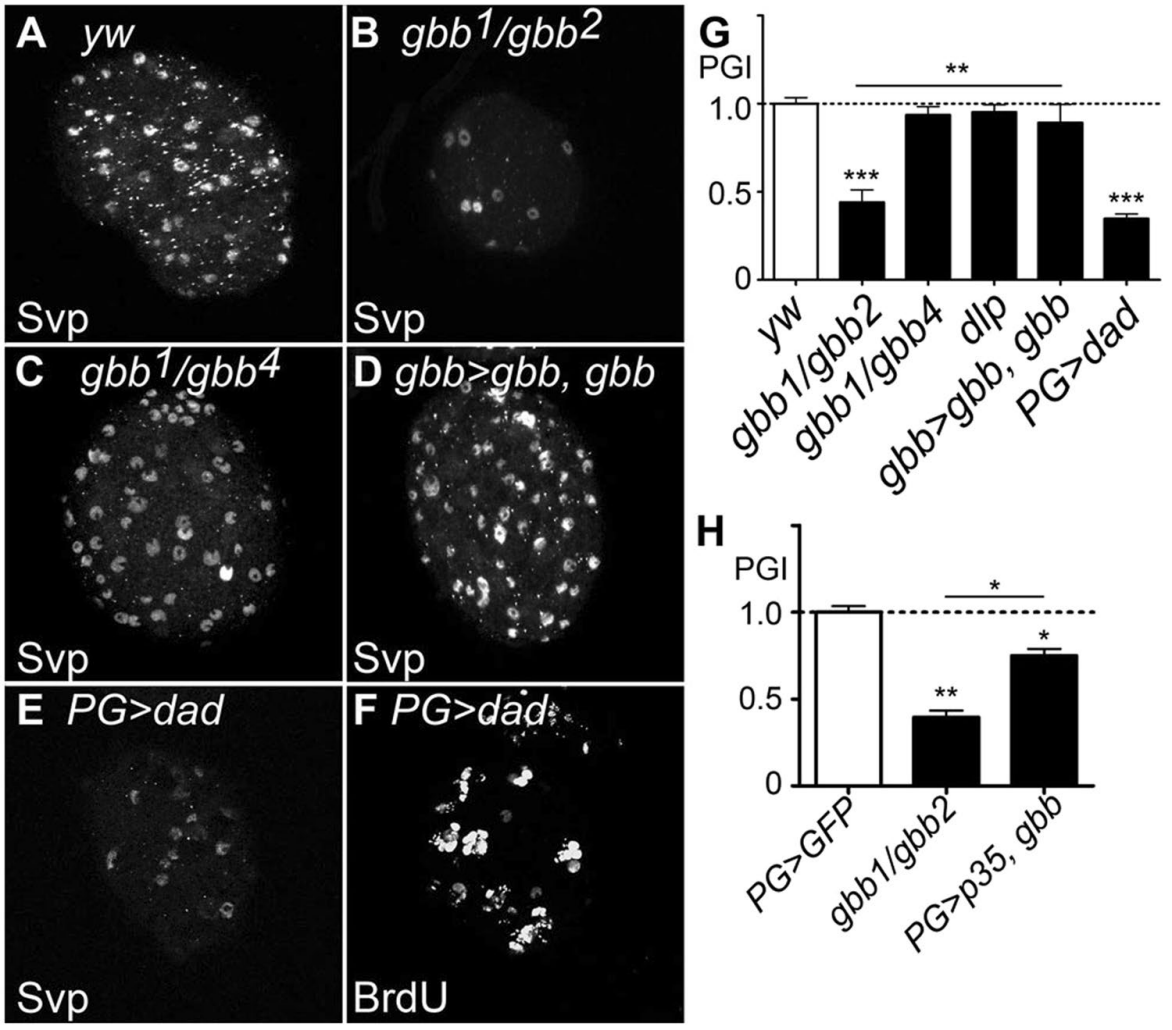
Function of Gbb paracrine signaling in PG maintenance. (**A**–**E**) Anti-Svp antibody staining of control (*yw* reared at 32 °C; **A**), *gbb*^*1*^*/gbb*^*2*^ (**B**), *gbb*^*1*^*/gbb*^*4*^ (reared at 32 °C; **C**), *gbb*^*1*^ *gbb-Gal4/gbb*^*2*^ *UAS-gbb* (**D**), and *PG-Gal4/UAS-dad* (**E**) brains. (**F**) BrdU incorporation in a *PG-Gal4/UAS-dad* brain. The number of Svp-positive PG was significantly reduced in *gbb*^*1*^*/gbb*^*2*^ but not in *gbb*^*1*^*/gbb*^*4*^ animals. PG number and NB proliferation were also diminished by induction of *UAS-dad* using *PG-Gal4*. (**G** and **H**) Summary of PG Index (PGI). To calculate PGI, the number of PG cells in brains of each genotype was divided by that of control brains (*yw* in **G**, *PG-Gal4*/UAS-*nlsGFP* in **H**). The number of PG cells was counted as Svp-positive cells (Svp, **G**) or GFP-positive cells in *PG-Gal4*/UAS-*nlsGFP* brains (GFP). The genotypes used in H are: *PG-Gal4*/UAS-*nlsGFP*, *PG-Gal4 gbb*^*1*^*/gbb*^*2*^*; UAS-nlsGFP*/+, and *PG-Gal4 gbb*^*1*^*/UAS-p35 gbb*^*2*^*; UAS-nlsGFP*/+. Numerical figures depict the mean ± SE. *p < 0.05; **p < 0.01.

**Figure 6:**
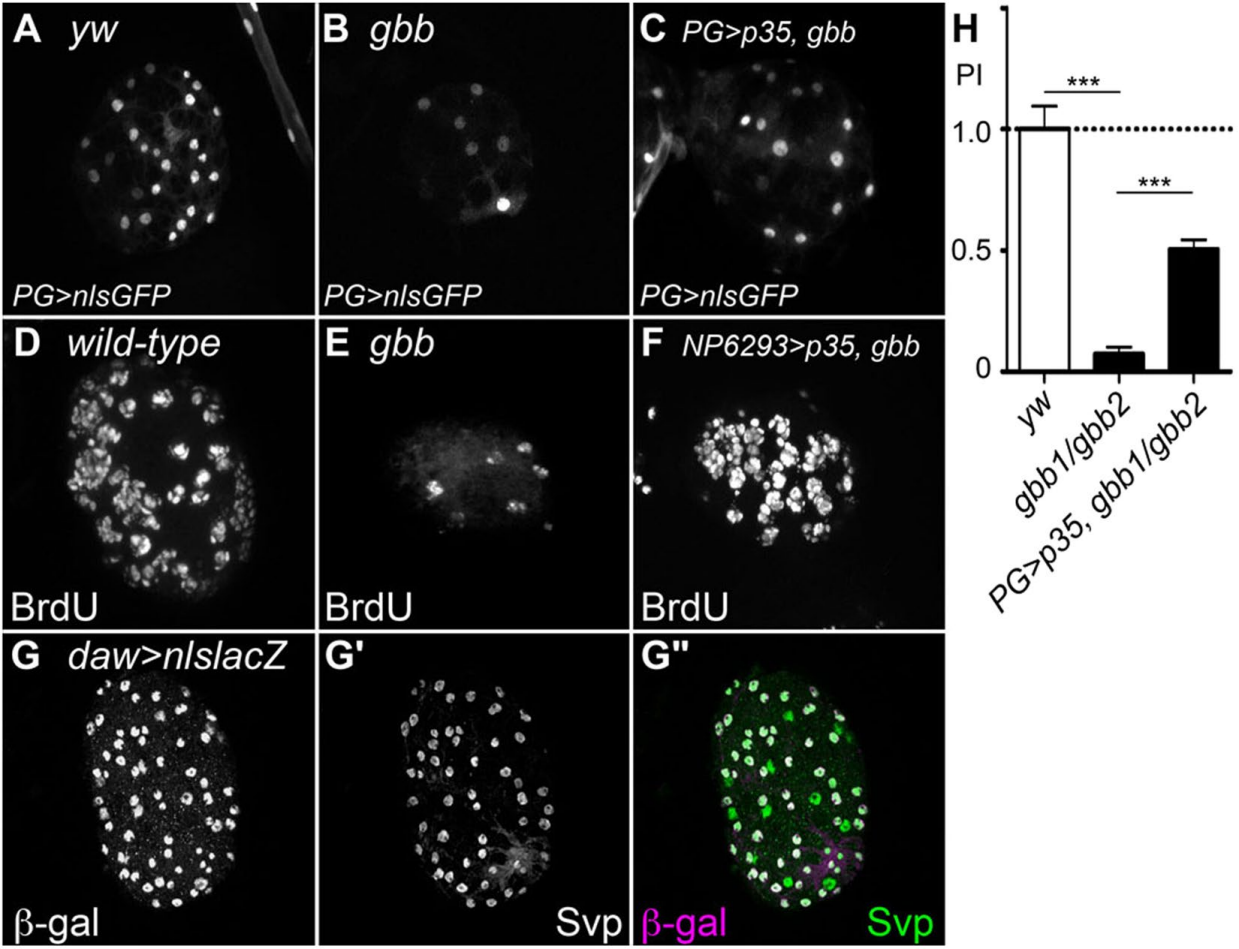
Rescue of PG in *gbb* null mutants by *UAS-p35* can restore NB proliferation. (**A–C**) PG marked by GFP expression in *PG-Gal4*/UAS-*nlsGFP* (**A**), *PG-Gal4 gbb*^*1*^*/gbb*^*2*^*; UAS-nlsGFP* (**B**), and *PG-Gal4 gbb*^*1*^*/UAS-p35 gbb*^*2*^*; UAS-nlsGFP* (**C**) brains. *p35* expression partially restored PG number. (**D**–**F**) BrdU incorporation in *yw* (**D**), *gbb*^*1*^*/gbb*^*2*^ (**E**), and *PG-Gal4 gbb*^*1*^*/gbb*^*2*^*; UAS-p35*/+ (**F**) brains. *p35* expression partially rescues the NB proliferation defect of *gbb* null mutants. (**G–G”**) *daw* is expressed in PG. A *daw* > *nlsLacZ* brain was stained with anti-β-galactosidase (**G**) and anti-Svp (**G’**) antibodies. (**G”**) Merged image of G and G’. *daw* is exclusively expressed in Svp-positive PG. (**H**) Summary of PI for each genotype. ***p < 0.001.

The observation that both NBs and PG receive Gbb signaling (monitored by *dad-lacZ*, Fig. 4A–B”) raised the question of what role this pathway plays in PG. Remarkably, we found that the number of PG cells, monitored by anti-Svp antibody staining, was substantially reduced in *gbb* null mutant brains (Figs 5B,G and 6B; PG Index (PGI) = 0.44). This phenotype is unique to the *gbb* null condition since *gbb* hypomorphic mutations did not affect PG cell numbers (Fig. 5C and G; PGI = 0.94). The loss of PG cells in *gbb* null mutants was rescued by *UAS-gbb* expression in NBs using *gbb-Gal4* (Fig. 5D and G; PGI = 0.89). Thus, in addition to NB proliferation, Gbb signaling regulates PG maintenance. The lack of this phenotype in *gbb*^*1*^*/gbb*^*4*^ and *dlp* mutants (Fig. 5G; PGI = 0.95) suggests that the critical threshold required for PG maintenance is lower than that for NB proliferation. Consistent with the phenotype of *gbb* null mutants, blocking BMP signaling in PG by *UAS-dad* expression using *PG-Gal4* also reduced the PG cell number (Fig. 5E and G; PGI = 0.35). This treatment also resulted in impaired NB proliferation (Figs 5F and 4K; PI = 0.32), consistent with the essential role of PG in the normal proliferation of NBs.

In order to determine if PG cells are lost by cell death in *gbb* null mutants, we expressed *p35*, an apoptosis inhibitor, in PG. In this experiment, we counted the PG cell number by marking PG with nlsGFP driven by *PG-Gal4*. We found that *p35* expression significantly restored the PG in *gbb* null mutant brains (Figs 6A–C and 5H). Altogether, these results indicate that Gbb acts not only as an autocrine factor in NB but as a paracrine survival signal to inhibit PG apoptosis.

Interestingly, in addition to rescuing PG, *p35* expression in *gbb* null mutants also partially restored NB proliferation (Fig. 6D–F and H; PI = 0.50). Given that this restoration occurs in the absence of Gbb function, this observation implies that additional factor(s) derived from PG promote NB division, independent of Gbb signaling. Possible candidates for such molecules are activin-like ligands since, as mentioned above, a previous study showed that mutations in *baboon*, the activin type I receptor, or *dSmad2*, the downstream activin signal transducer, strongly disrupt NB proliferation^6^. We examined the expression pattern of one of the *Drosophila* activin-like ligands, *daw*, in the CNS. Extensive co-localization of Svp and *daw-Gal4* expression indicates that this ligand is exclusively expressed in PG (Fig. 6G–G”). This result is consistent with the idea that Daw, and possibly other activin-like ligands, serve as PG factors to control NB proliferation.

Since both *dlp* and activin signaling components show NB proliferation defect, we asked if they function in the same pathway (i.e. Dlp acts as a co-receptor for activins). We examined the genetic interactions between *babo* and *dlp* for brain growth using the PR projection assay. No synergistic effect was observed between *babo* and *dlp* mutations (data not shown), suggesting that activins regulate NB proliferation, independent of Gbb-Dlp signaling.

### Possible mechanisms for communications between perineural glia and NBs

Our results showed that PG and NBs communicate to regulate each other’s development. Specifically, our findings support the idea that Dlp serves as a co-receptor for Gbb signaling during larval brain development. However, the mechanism by which these cells communicate across SPG, the major barrier component of the BBB, is unknown. One obvious possibility is that “transcytosis” in SPG may carry specific molecules, either alone or in complexes with lipid particles, to the other side of the BBB^60^. Alternatively, SPG may have unknown openings, which allow PG to directly contact NBs. Although we do not know the definite answer to this question, several observations described below are consistent with these possibilities.

**Figure 7:**
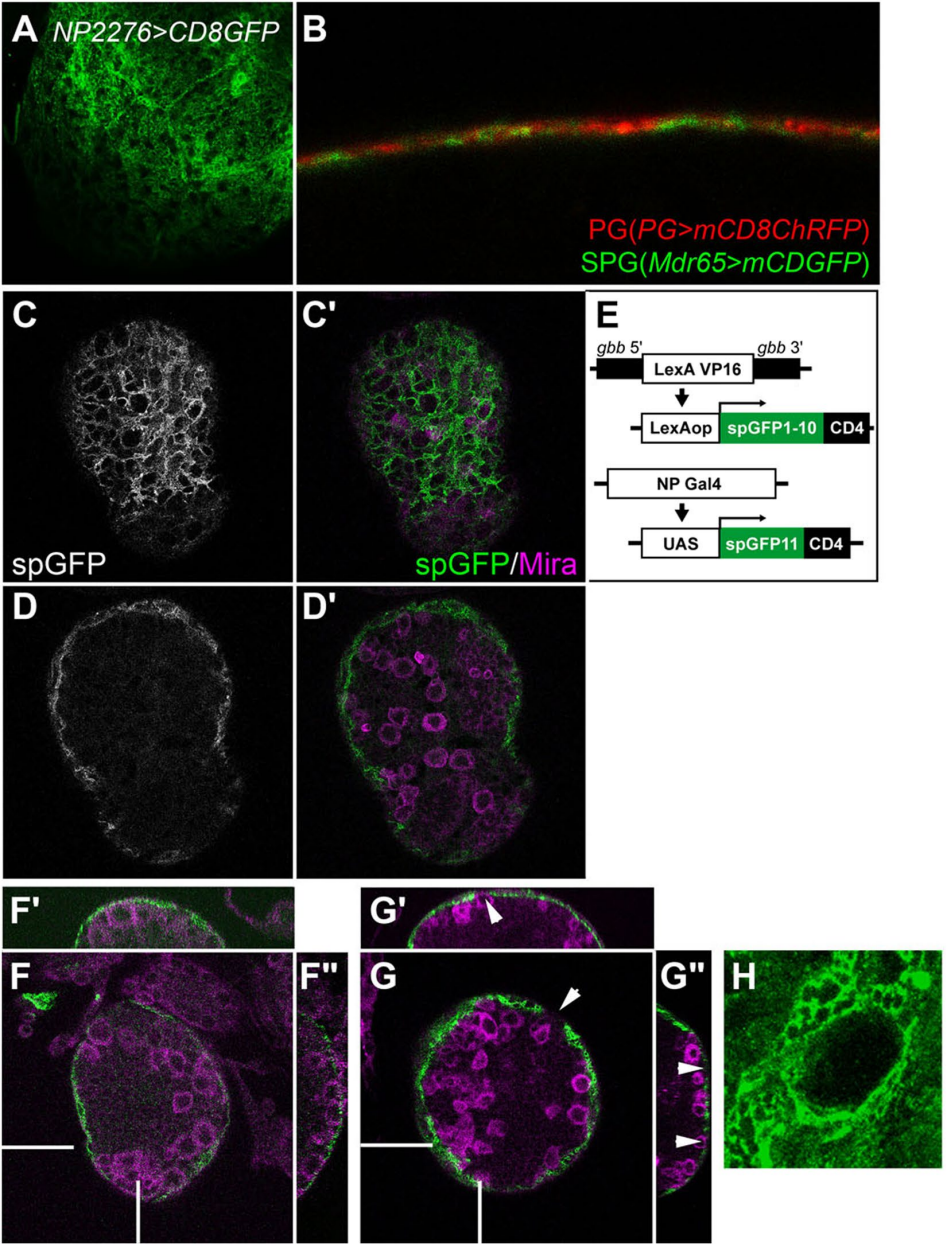
Possible membrane contact between NBs and PG. (**A**) Distribution of a membrane-bound form of GFP in SPG (*SPG* > *mCD8GFP*). (**B**) Cross-sections of brains doubly stained for PG (*PG-Gal4* > *UAS-mCD8ChRFP*) and SPG (*Mdr65-LexA* > *mCD8GFP*). (**C–D’**) Reconstitution of spGFP fragments. spGFP signal was detected in *PG-Gal4/gbb-LexAVP16; UAS-spGFP11/LexAop-spGFP1-10* brains in the glial cell focal plane (**C** and **C’**) and NB focal plane (**D** and **D’**). Anti-Mira signal is shown in C’ and D’ (magenta). (**E**) Schematic representation of spGFP reconstitution. *spGFP1-10* was expressed in NBs under the control of the *gbb* promoter through the LexA/LexAop system. *spGFP11* expression was driven in the PG by NP Gal4 drivers. (**F**–**G**”) *shi*^*ts*^ expression disrupts the PG-NB contact. spGFP signal (green) in PG-ablated brains (*PG-Gal4/gbb-LexAVP16; UAS-spGFP11/LexAop-spGFP1-10, UAS-shi*^*ts*^) at permissive (**F–F”**) and restrictive (**G–G”**) temperatures. Anti-Mira staining marks NBs (magenta). Images for X-Z sections (**F’** and **G’**) and Y-Z sections (**F”** and **G”**) at white bars are shown. Arrows show the lack of GFP signal, showing the elimination of physical contact between PG and NBs (**G**). An example is shown of a large gap in the spGFP signal on the brain surface (**H**).

When a membrane-bound form of GFP (mCD8GFP) was expressed in SPG by the *SPG-Gal4* driver, GFP signal was detected non-uniformly, showing openings within the SPG layer (Fig. 7A). These numerous holes suggest either the presence of a zone where mCD8GFP is excluded from the membrane or a discontinuity of the plasma membrane. Cross-sections of brains doubly stained for PG (*PG-Gal4* > *UAS-mCD8ChRFP*) and SPG (*Mdr65-LexA* > *mCD8GFP*) often revealed regions in which the PG and SPG signals are found in the same layer (Fig. 7B). They do not appear to be co-localized since we do not see yellow signals indicative of co-localization. Instead, in these domains, the SPG layer is undetectable and the only PG signal can be detected (Fig. 7B). Such discontinuity of the SPG signal is consistently observed in virtually all brain samples analyzed. To further confirm that this staining pattern is not due to leaky expression of *PG-Gal4* in SPG, we used eGFP-tagged Dlg, which is localized to septate junctions formed between neighboring SPGs. When this construct was expressed in SPG using *Mdr65-Gal4*, eGFP-Dlg highlighted the septate junction structures (Fig. S1E). On the other hand, eGFP-Dlg did not show this pattern when expressed by *PG-Gal4* or *daw-Gal4*. Thus, these two Gal4 drivers are specific to PG and do not drive expressions in SPG. Together, our observations suggest that the PG layer may directly communicate with more interior NBs through the openings in the SPG.

To examine whether there is close physical proximity of NBs and PG cell membranes, we employed the split-GFP (spGFP) system. This method is based on the fact that complementation of two split-GFP proteins, spGFP1-10 and spGFP11, reconstitutes green fluorescence while each spGFP fragment alone is not fluorescent^61^. Membrane-bound forms of spGFP proteins have been successfully used to label areas of close membrane apposition between two cells^62^. We developed a system to express each spGFP fragment separately in different set of cells *in vivo* using the Gal4/UAS^63^ and the LexA/LexAop^64^ systems. We generated and used *gbb-LexA* as a NB driver to express a membrane-tethered form of spGFP1-10 in NBs (Fig. 7E). Independent of spGFP1-10, expression of spGFP11 was induced in several different types of glial cells using the Gal4/UAS system. In addition to PG-specific *PG-Gal4*, we used *NP2222-Gal4* and *NP3233-Gal4* to induce expression of a membrane bound form of spGFP11 in cortex glia and astrocyte-like glia, respectively^38,65^.

Using PG-driven spGFP11, we detected complementation of the two spGFP fragments, suggesting that PG cell membranes are in close proximity to NB membranes (Fig. 7C and D). Control brains lacking one of the transgenes showed no GFP signal, proving the specificity of the GFP reconstitution (Fig. S4A–D). Similar experiments with other Gal4 drivers showed that cortex glia have large area of direct contact with NBs (Fig. S4F’), while astrocyte-like glia show very limited contact with NB neuropil (Fig. S4H’). These are expected results based on the anatomy of the larval CNS, further validating the spGFP experiments to detect the membrane contact. Notably, PG and cortex glia showed distinct patterns of spGFP signals: cortex glia contact the entire surface of a NB, while PG contact only the apical membrane of NBs (Figs 7C,D’ and S4), suggesting that each glial cell population may have a unique contribution to NB development.

We also examined NB-PG membrane contact by the spGFP system in PG > *shi*^*ts*^ brain, which shows severely reduced NB proliferation (Fig. 2). At 18 °C, GFP complementation was observed uniformly and continuously on the surface of the brain lobe although the GFP signal is weak due to the low level of Gal4-induced expression at low temperature (Fig. 7F–F”). In contrast, inactivation of *shi* at the restrictive temperature caused gaps in the spGFP signal, indicating loss of the PG-NB contact on the brain lobe surface (arrowheads in Fig. 7G–G”). Figure 7H shows an example of a large gap in the spGFP signal on the surface of a *shi*^*ts*^ brain lobe at 32 °C. These observations suggest that the NB proliferation defect in *shi*^*ts*^ brain may be caused by loss of contact-dependent communication between NB and PG due to disrupted membrane trafficking in PG.

Taken together, our observations using the spGFP complementation system suggest that PG-NB communication may be mediated through direct membrane contact or transcytotic transport of membrane components across the BBB.

## Discussion

Glial cells play essential roles in the *Drosophila* neural stem cell niche^66,67^. Our study demonstrated that cellular communication between NBs and surface glia is critical for development of both cell types. Gbb signaling is required autonomously for the proliferation of NBs as well as non-autonomously for the survival of PG. PG is essential for Gbb autocrine signaling. Given that the NB phenotype in *PG* > *shi*^*ts*^ is associated with aberrant subcellular localization of Dlp in PG (Fig. 2L), one contribution of PG appears to be presenting Dlp on their surface, which likely potentiates Gbb autocrine signaling.

Alignment of amino acid sequences of *Drosophila* BMPs identified an HS-binding domain in Dpp and Gbb, but not Screw^68^. A truncated form of Dpp lacking this HS-binding fails to bind to its co-receptor Dally and was quickly degraded by receptor-mediated endocytosis^69^. This suggested that glypicans can enhance BMP signaling by disrupting receptor-mediated internalization and degradation of the Dpp-receptor complex. We have also shown that both Dally and Dlp can act as a trans co-receptor to enhance Gbb signaling *in vitro*^11^.

Expression of p35 by *PG-Gal4* in *gbb* null mutants not only rescued PG but also partially restored NB proliferation. This observation suggests that additional PG-derived factor(s) contribute to NB proliferation. The most likely candidates for these PG factor(s) are activin-like ligands, whose role in NB division has been previously reported^6^. We identified specific expression of Daw in PG, supporting the idea that activin signaling together with Gbb regulates NB proliferation. Altogether, glia play a “niche-like” role, contributing to the microenvironment required for normal development of contacting NBs. Glia provide Dlp to support Gbb autocrine signaling, and express activin-like ligands as additional promoters of NB proliferation. Thus, we propose a model that bidirectional communication between NBs and glia through TGF-β signaling pathways influences each other’s development in the NSC niche.

Our study raises a fundamental question: how can PG and NBs communicate across the BBB and affect each other’s development? Our experiments using spGFP complementation favor a model in which NBs make direct contact with PG. Thus, it is possible that Dlp acts as a trans co-receptor for Gbb at the contacting sites. However, our study does not exclude other possibilities. For example, one can expect that transcytosis in SPG would allow molecular communications across this layer. Thus, the spGFP complementation signals we observed may result from the transport of membrane components of PG and NBs. It has been shown that lipoprotein particles can cross the *Drosophila* BBB^60^. The major *Drosophila* lipoprotein, lipophorin (Lpp), forms large particles with lipid molecules and transports them between different organs. In addition to lipid, Lpp particles carry sterol-linked and GPI-linked proteins. Dlp is a GPI-linked HSPG and has been shown to be incorporated into the Lpp particles^70^. This raises an interesting possibility that Dlp is transcytosed by the SPG through a Lpp-mediated mechanism.

Recent studies have shown that the surface glia are required to maintain the structural integrity of the neural lamella and to control the shape of the CNS^71^. A transcriptome study demonstrated that the surface glia express metabolic enzymes and signaling molecules at high levels in addition to various transporters, cell adhesion molecules, and ECM components^72^. Our study also supports the idea that the surface glia play a role not only as a simple structural insulators but a dynamic regulator of brain physiology development. Further study will be required, to fully elucidate the cellular and molecular mechanisms by which surface glia regulate the microenvironment internal to the BBB.

## Methods

### Fly stocks

Detailed information for the fly strains used is described in Flybase (http://flybase.bio.indiana.edu/) except where noted. Fly strains used were: *dlp*^*1* ^^15^, *dlp*^*A187 *^^73^, *gbb*^*1* ^^28^, *gbb*^*2*^, *gbb*^*4*^, *dally*^*gem *^^21^, *ttv*^*524* ^^74^, *babo*^*Fd*4 ^^33^, *pros-Gal4* (a gift from F. Matsuzaki), four Gal4 drivers for glial cells: *NP6293-Gal4* (*PG-Gal4*), *NP2276-Gal4* (*SPG-Gal4*), *NP3233-Gal4*, and *NP2222-Gal4*^38,65^, *daw-Gal4*^6^, *gbb-Gal4*, *Mdr65-LexA* (Bloomington Stock Center, BL61562), *UAS-lacZ*, *UAS-dlp39.2*^15^, *UAS-p35* (Bloomington Stock Center), *UAS-dad*^58^, *UAS-gbb*^75^, *UAS-mCD8GFP*^76^, *UAS-ManII-eGFP*^77^, *UAS-shi*^*ts* ^^48^, *UAS-nlsGFP*, *UAS-eGFP-Dlg* (a gift from V. Budnik), *LexAop-rCD2::GFP*^64^, *LexAop2-mCD8GFP* (Blooming Stock Center, BL32203), *vkg*^*G454*^ (*viking::GFP*)^78^, *gbb-lacZ*, *dad-lacZ*^58^, and *UAS-mCD8ChRFP*. Plasmid DNAs for *gbb-LexAVP16*, *LexAop-spGFP1-10*, and *UAS-spGFP11* were constructed as described below and transgenic strains bearing these constructs were generated by Rainbow Transgenic Flies, CA.

For overexpression experiments using the Gal4/UAS system, flies were raised at 25 °C for 24 hours AEL and then incubated at 29 °C until the mid-third instar larval stage. To compromise temperature sensitive alleles, flies bearing either *UAS-shi*^*ts*^ or *gbb*^*4*^ were exposed to temperature shift from 18 °C to 32 °C with water-bath until the stage we examined. Larvae were staged by counting the number of teeth on the mouth hook. Homozygotes and trans-heterozygotes were selected based on GFP expression from the *CyO-GFP* balancer or the *Tb* dominant marker of *TM6B*.

### Immunostaining and imaging

The following antibodies were used: Guinea pig anti-β-galactosidase (1:500, a gift from Y. Hiromi), rat anti-β-galactosidase (1:500, a gift from Y. Hiromi), rabbit anti-β-galactosidase (1:500, Cappel), mouse anti-Repo (1:200, Developmental Studies Hybridoma Bank (DSHB) 8D12), mouse anti-Chaoptin (1:100, DSHB, 22B10), rat anti-Miranda (Mira) (1:100), guinea pig anti-Deadpan (Dpn) (1:1000), mouse anti-BrdU (1:250, Becton, Dickinson and Company), mouse anti-Dlp (1:4, DSHB, 13G8), rabbit anti-active caspase 3 (1:500, Abcam), mouse anti-Svp (1:4, a gift from Y. Hiromi), rabbit anti-pH3(1:1000, Upstate Biotechnology), and Alexa Fluor 488, 546, 633 conjugated secondary antibodies (1:500, Invitorgen).

Larval brains were dissected in PBS and fixed with 3.7% paraformaldehyde in PBS for 30 min at room temperature. After washing with PBST (0.1% triton X-100 in PBS), tissues were blocked with 5% normal goat serum in PBST for 30 min. The samples were incubated with primary antibodies at 4 °C overnight. After washing with PBS, the samples were incubated with secondary antibodies for 2 hours at room temperature. The tissues were mounted in Vectashield (VectaLabs) and fluorescent signals were observed by confocal laser scanning microscope (Zeiss LSM710) with a 40X water immersion objective.

#### BrdU incorporation

Larval brains were dissected in PBS and incubated with Schneider’s *Drosophila* medium (Invitrogen) containing 40 ng/ml of BrdU (Roche) for 30 min at room temperature, and subsequently with Schneider’s *Drosophila* medium without BrdU for 24 hours. Tissues were washed in PBST and fixed with 3.7% paraformaldehyde in PBT for 30 min at room temperature. After tissues were treated with 1 N HCl for 30 min at room temperature, immunostaining was performed as described above. Proliferation Index (PI) was calculated as follows. The number of BrdU positive neurons from NBs in experimental brains was divided by that of wild-type control brains (*yw* or Oregon R). The control brains were labeled with BrdU in the same tube with experimental brain samples and the genotypes were distinguished by *y*^+^ or *y*^−^ mouth hook.

#### *In vitro* characterization of Gbb

*Drosophila* S2 cells were grown in Schneider’s medium (Invitrogen) supplemented with 10% FBS, 100 U/ml penicillin and 100 mg/ml streptomycin. Transient transfections were performed using Effectene (Qiagen) as per manufacturer’s instructions.

There are multiple proteolytic processing sites in Gbb, which produce distinct mature ligand forms, including *gbb*^*1*^5 and Gbb38, and the amount of each product expressed in S2 cells is generally low. When a few cleavage sites are blocked, a larger amount of Gbb38 is produced^59^. S2 cells were transfected with pAW-Gbb[mS1mS0]-HA and incubated for 72 hours. The conditioned medium containing Gbb38-HA was incubated with heparin sepharose CL-6B (Amersham Biosciences) for 4 hours. After extensive washing of unbound proteins, a heparin-bound fraction was eluted by 0.25 M NaCl and analyzed by immunoblotting using anti-HA antibody.

For cellular immuno-colocalization analysis, S2 cells expressing Dlp-Myc and Gbb-HA were incubated with anti-HA and anti-Myc in a Concanavalin A-coated coverslip for 30 minutes at 25 °C, washed, and fixed with 3.7% formaldehyde. Dlp-Myc and Gbb-HA were detected as described before^79^.

### Plasmid construction

The original plasmids of spGFP fragments, *ace-4p CD4-2 spGFP1-10* and *rig-3p CD4-2 spGFP11*, were provided by C. Bargmann^62^. To optimize the spGFP constructs for *Drosophila*, the original signal peptide (Par3) was replaced with the *Drosophila myospheroid* signal peptide (1-28). The spGFP11 and spGFP1-10 fragments were amplified by PCR using primers 1 and 3, and primers 2 and 3, respectively (see below), and subcloned into the pENTR/D-TOPO Gateway entry vector (Invitrogen). PCR fragments were verified by DNA sequencing. The destination vectors used for germline transformation were pTW from the *Drosophila* Gateway Vector Collection and pLot W from S. Diegelmann^80^.

*gbb-gal4* and *gbb-lacZ* constructs were made by ligating *gal4* and *lacZ* sequences downstream of the 5′ genomic sequences of the *gbb* gene, respectively, and subcloned into pCaSpeR3. *gbb-LexAVP16* was constructed in the same manner after the *LexAVP16* fragment was amplified by PCR using primers 4 and 5.

**Primer 1**: 5′-CACCATGATCCTCGAGAGAAACCGGAGGTGCCAGCTGGCCCTCCTCATGATCGCAATACTGGCCGCCATCGCTGGACAAACGGATGCCCAGCGTGACCACATGGTCCTTCATGAGTATGTAAATGC-3′

**Primer 2**: 5′-CACCATGATCCTCGAGAGAAACCGGAGGTGCCAGCTGGCCCTCCTCATGATCGCAATACTGGCCGCCATCGCTGGACAAACGGATGCCCAGATGTCCAAAGGAGAAGAACTGTTTACC-3′

**Primer 3**: 5′-CTAGCGCCTTCGGTGCCGG-3′

**Primer 4**: 5′-GTGGCGGCCGCTCCGTCCAAAACACGAAATAATGAAAGCGTTAACGGCCAGGCAACAAGAG-3′

**Primer 5**: 5′-GTGGCGGCCGCCTACCCACCGTACTCGTCAATTCCAAGGGC-3′

### Data availability

All data generated or analyzed during this study are included in this published article and its Supplementary Information files.

## Electronic supplementary material

Supplemental Information
