# Supplementary material for: Regulation of neuroblast proliferation by surface glia in the Drosophila larval brain
Source: Sci Rep. 2018 Feb 27;8:3730. doi: 10.1038/s41598-018-22028-y (PMC5829083; doi:10.1038/s41598-018-22028-y)
Supplement: Supplementary file 1 — Supplemental Information [file 41598_2018_22028_MOESM1_ESM.pdf]

Supplemental Information

Regulation of neuroblast proliferation by surface glia in the *Drosophila* larval brain

Makoto I. Kanai§, Myung-Jun Kim§, Takuya Akiyama, Masahiko Takemura, Kristi Wharton, Michael O'Connor, and Hiroshi Nakato

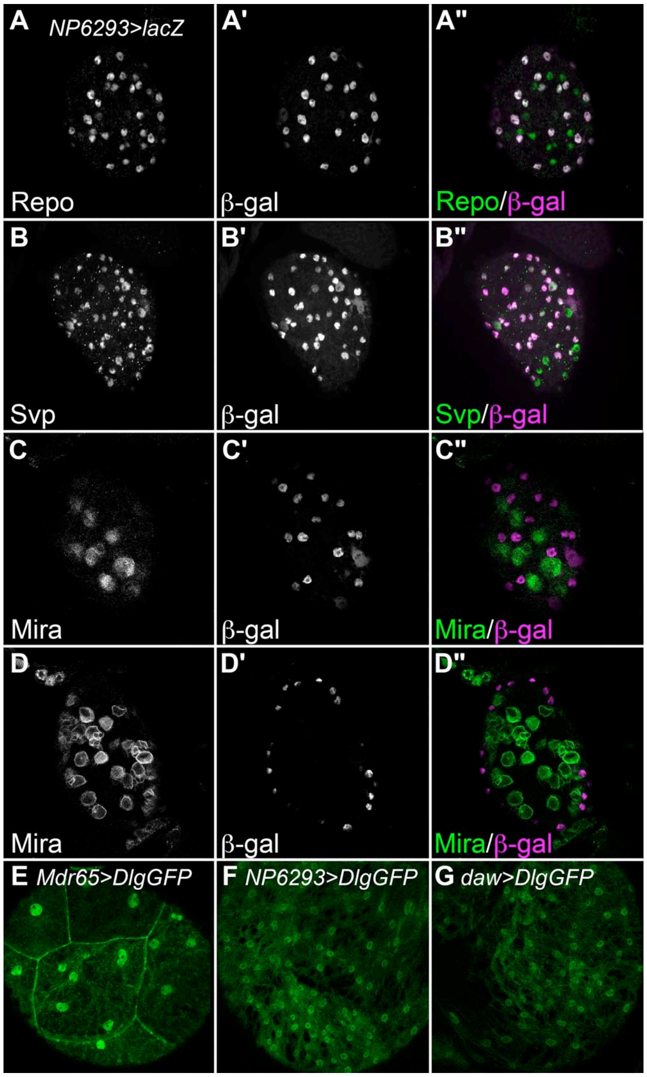

Fig. S1. Expression of *NP6293-Gal4* in the *Drosophila* brain.

(A-A") *NP6293-Gal4* (*PG-Gal4*) is expressed in a subset of glial cells (PG) in the *Drosophila* brain. A *NP6293>lacZ* brain was stained with anti-Repo (A) and anti-β-galactosidase (A') antibodies. (A") Merged image of A and A'. (B-B") Seven-up (Svp) is expressed in the PG. A *NP6293(PG)>lacZ* brain was stained with anti-Svp (B) and anti-β-galactosidase (B') antibodies.

(B") Merged image of B and B'. (C-D") A *NP6293(PG)>lacZ* brain was stained with anti-Mira (C and D) and anti-β-galactosidase (C' and D') antibodies. C-C" and D-D" show the surface glia- and NB-focal planes, respectively. C" and D" are merged images of the two left images. The anti-β-galactosidase signals (C' and D') were not co-localized with anti-Mira, a marker of NB (C and D), showing that *PG-Gal4* is not expressed in NBs. (E-G) Expression of a Disc-large-GFP fusion protein (DlgGFP) in SPG by *Mdr65-Gal4* marks septate junctions at the periphery of SPG cells (E). DlgGFP expression by *NP6293-Gal4* (F) or *dawdle-Gal4* (G) does not show septate junction staining, further confirming PG-specific expression of these two drivers.

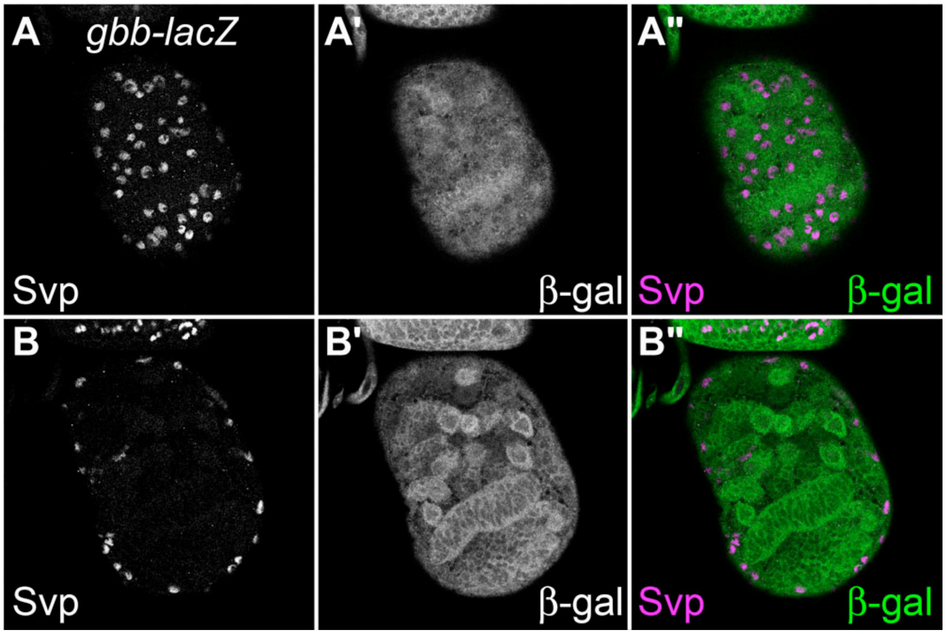

Fig. S2. ***gbb-lacZ* expression in the larval brain.**

A *gbb-lacZ*/+ brain was stained with anti-Svp (A and B) and anti-b-galactosidase (A' and B') antibodies. A-A" and B-B" show the surface glia- and NB-focal planes, respectively. A" and B" are merged images of the two left images. *gbb-lacZ* signal is not co-localized with anti-Svp, a marker for PG.

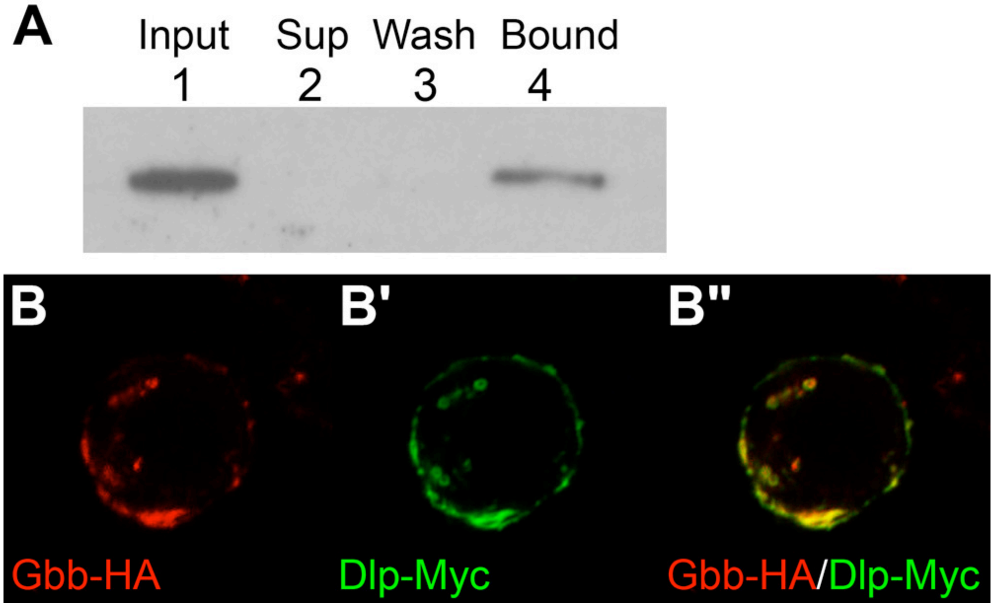

Fig. S3. In vitro characterization of Gbb

(A) Gbb is a heparin-binding protein. Gbb-HA was expressed in S2 cells and the conditioned medium was incubated with heparin sepharose CL-6B beads for 4 hours. After extensive washing of unbound proteins, a heparin-bound fraction was eluted by 0.25 M NaCl. The conditioned medium (lane 1), supernatant after the incubation with heparin-sepharose (lane 2), unbound proteins in washing solution (lane 3), and the eluate (lane 4) were analyzed by immunoblotting with anti-HA antibody. (B) Gbb-HA (red) and Dlp-Myc (green) show extensive colocalization on the surface of an S2 cell. In this protocol, the cells were incubated with anti-HA and anti-Myc antibodies before permeabilization and fixation. Therefore, the antibodies detect only extracellular antigens.

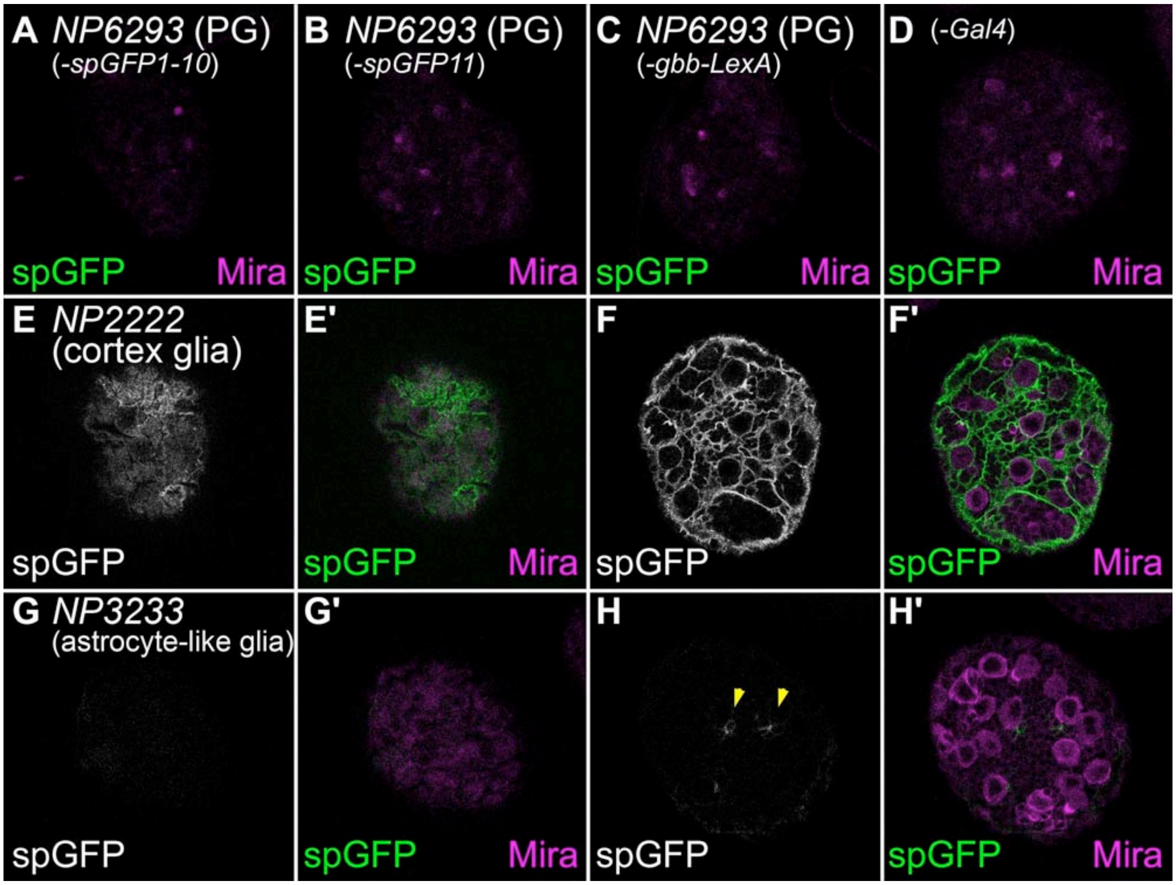

Fig. S4. Contact patterns of NBs with cortex glia and astrocyte-like glia.

(A-D) Negative controls for spGFP assay using *PG-Gal4*. spGFP signals were not detected in *PG-Gal4/gbb-LexAVP16; UAS-spGFP11/+* (*-spGFP1-10*; A), *PG-Gal4/gbb-LexAVP16; +/LexAop-spGFP1-10* (*-spGFP11*; B), *PG-Gal4/+; LexAop-spGFP1-10/UAS-spGFP11* (*-gbb-LexA*; C), and *+/gbb-LexAVP16; UAS-spGFP11/LexAop-spGFP1-10* (*-Gal4*; D) brains. (E-F') spGFP signals showing cortex glia-NB interaction. Robust GFP signals were observed on the surface of NBs in *NP2222-Gal4/gbb-LexAVP16; UAS-spGFP11/LexAop-spGFP1-10*. (G-H') spGFP signals in *gbb-LexAVP16 LexAop-spGFP1-10; NP3233-Gal4/UAS-spGFP11*. Anti-Mira antibody staining marks NBs (A', A''', B', and B'''). Limited contact was detected between astrocyte-like glia and NBs (arrows in H). Confocal images are shown for glial (brain surface) focal planes (E, E', G, and G') and NB focal planes (F, F', H, and H').
